# Recycling of phenolic compounds in Borneo’s tropical peat swamp forests

**DOI:** 10.1186/s13021-018-0092-6

**Published:** 2018-02-07

**Authors:** Catherine M. Yule, Yau Yan Lim, Tse Yuen Lim

**Affiliations:** 1 Present Address: School of Science and Engineering, University of the Sunshine Coast, Sippy Downs, QLD 4556 Australia; 2 Tropical Medicine and Biology Multidisciplinary Platform, School of Science, Monash University, Jalan Lagoon Selatan, 47500 Bandar Sunway, Selangor Malaysia

**Keywords:** Tannins, Flavonoids, Phenolic recycling, *Macaranga pruinosa*, Malaysia

## Abstract

**Background:**

Tropical peat swamp forests (TPSF) are globally significant carbon stores, sequestering carbon mainly as phenolic polymers and phenolic compounds (particularly as lignin and its derivatives) in peat layers, in plants, and in the acidic blackwaters. Previous studies show that TPSF plants have particularly high levels of phenolic compounds which inhibit the decomposition of organic matter and thus promote peat accumulation. The studies of phenolic compounds are thus crucial to further understand how TPSF function with respect to carbon sequestration. Here we present a study of cycling of phenolic compounds in five forests in Borneo differing in flooding and acidity, leaching of phenolic compounds from senescent *Macaranga pruinosa* leaves, and absorption of phenolics by *M. pruinosa* seedlings.

**Results:**

The results of the study show that total phenolic content (TPC) in soil and leaves of three species of *Macaranga* were highest in TPSF followed by freshwater swamp forest and flooded limestone forest, then dry land sites. Highest TPC values were associated with acidity (in TPSF) and waterlogging (in flooded forests). Moreover, phenolic compounds are rapidly leached from fallen senescent leaves, and could be reabsorbed by tree roots and converted into more complex phenolics within the leaves.

**Conclusions:**

Extreme conditions—waterlogging and acidity—may facilitate uptake and synthesis of protective phenolic compounds which are essential for impeded decomposition of organic matter in TPSF. Conversely, the ongoing drainage and degradation of TPSF, particularly for conversion to oil palm plantations, reverses the conditions necessary for peat accretion and carbon sequestration.

## Background

Indo-Malaysian tropical peat swamp forests (TPSF) sequester about 10% of total global peatland carbon, and 65% of tropical peatland carbon [1–3]. Carbon in TPSF is stored largely in the form of phenolic compounds. Most of the carbon is in the form of lignin derived from trees up to 70 m tall, and the products of lignin degradation, in layers of peat up to 20 m deep. The colour of the blackwaters that saturate the substrate and seasonally flood TPSF is due to tannins, humic acids and other phenolic compounds that leach from leaf litter. Other phenolics common in TPSF are the plant secondary compounds that protect the vegetation from herbivory, pathogen attack and UV damage and that confer colour to flowers, fruits etc. [4–9]. Abundance of phenolic compounds (total phenolic content—TPC—and tannins) varies seasonally and spatially with peat depth, within different plant species, within different plant structures, and between plants growing on peat versus mineral soils [6, 9, 10]. Levels of TPC and tannins are significantly higher at the peat surface where newly senescent leaves fall than in deeper peats [10]. The concentration of phenolic compounds has been found to be higher in ferns, gingers and six species of *Macaranga* trees found in Malaysian TPSF compared with the same species growing on dry land mineral soils [6, 9]. Phenolic compounds are also more abundant in the peat substrate of pristine TPSF compared with degraded peats [6]. As TPSF are increasingly drained, selectively logged then clear felled, and finally subject to fire, levels of TPC and tannins progressively decrease in the remaining peat. TPC also varies within plants, being most abundant in the mature leaves of a common TPSF tree *Macaranga pruinosa* compared with young or senescent leaves, branches, trunks or buttresses. Furthermore, relative concentrations of low (phenolic acids, flavonoids) to high (tannins and derivatives) molecular weight phenolics decrease as leaves mature indicating conversion within leaves [10]. TPC and tannins also vary temporally, being significantly more abundant in surface peat and in mature leaves during the wet season compared with the dry season [10], suggesting that waterlogging influences the uptake or production of phenolic compounds by plants.

The peat soil of pristine TPSFs is permanently waterlogged and the forests flood during the wet season up to 50 cm or more above the forest floor. When plants die or senescent leaves fall, phenolic compounds rapidly leach out into the soil and water [11, 12] where they have been shown to have a major influence on organic matter decomposition and nutrient cycling [4, 13]. In TPSF the high levels of phenols in the plant litter—adaptations to reduce herbivory in the nutrient poor environment—are responsible for the acidic (pH 2.9–4.5), toxic conditions of the water and the peat substrate [11]. Although tannins in plant detritus can be used as a carbon source by soil microbes [14, 15] they can also have toxic effects as they can bind soil proteins and exoenzymes and also inhibit fungal respiration and nitrification and thus they can inhibit decomposition [4] and hence constrain nutrient cycling which is further inhibited by the anaerobic, acidic, nutrient poor environment. Consequently, since water only flows out of pristine TPSF (unlike nutrient rich freshwater swamp forests), the only source of new nutrients is from atmospheric deposition and so the nutrients cycle between the living biomass and the peat soil [8, 12].

Clearly high concentrations of phenolic compounds, which are characteristic of a wide range of TPSF plants, are adaptations to survive in the extreme, acidic, toxic, nutrient poor environment. Observations made during our previous studies regarding the distribution of phenolic compounds within plants and within their environment [6, 9, 10] provided some intriguing results which suggested the following hypotheses:

1. Observation: TPSF plants (including ferns, gingers and trees) had higher concentrations of phenolic compounds than the same species found on dry land mineral soils or degraded peat soils (e.g. drained, logged, burnt) [6, 9].
  Hypothesis: TPSF plants can absorb phenolic compounds from their environment and/or they are capable of synthesizing phenolic compounds.
2. Observation: Fine roots (functioning in water and nutrient uptake) of *M. pruinosa* had higher TPC than coarse roots (used for transport) [10].
  Hypothesis: Fine roots can absorb phenolic compounds from the phenolic rich peat water.
3. Observation: Mature leaves of *M. pruinosa* had the highest TPC concentrations compared with young or senescent leaves and other plant parts and furthermore, the relative concentrations of low molecular weight phenolics (phenolic acids and flavonoids) to high molecular weight phenolics (tannins and their derivatives) decreased as the leaves matured [10];
  Hypothesis: Young leaves are either capable of producing low molecular weight simple phenolic compounds (phenolic acids and flavonoids) and/or the plants absorb these from the peat substrate. Following this, mature leaves are capable of utilizing phenolic acids and flavonoids to synthesize high molecular weight phenolic compounds (tannins and their derivatives).

To investigate these hypotheses and gain a greater understanding of the role of phenolic compounds in tropical peat swamp ecosystems this study investigates the following questions using a field study comparing common trees in forested peat swamp and non-peat swamp sites in Malaysia and also two laboratory experiments:

- Study 1: Do phenolic compounds in the leaves of three common *Macaranga* species differ among different forest types subject to varying waterlogging and acidity (TPSF, freshwater forest, flooded limestone forest, dry limestone forest and secondary forest)?
- Study 2: Are senescent leaves a potential source of fresh phenolic compounds in TPSF? What are the dynamics of leaching of total phenolic content (TPC), total flavonoid content (TFC) and total tannin content (TTC) from *M. pruinosa* leaves?
- Study 3: Is *M. pruinosa* capable of directly absorbing phenolic acids and flavonoids from peat water or are they solely synthesized within the plants?

## Methods

### Study sites

**Fig. 1:**
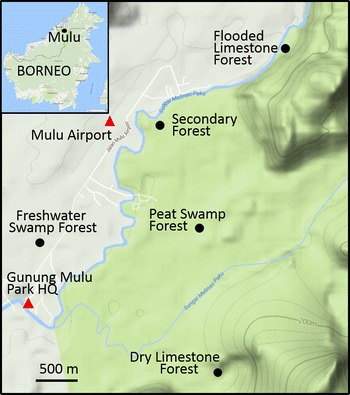
Location of study sites in Mulu National Park, Sarawak, Malaysia

Five study sites in Sarawak (East Malaysia—Borneo) were selected for Study 1 to investigate phenolic compounds in the leaves of three *Macaranga* species among different forest types: TPSF, freshwater forest, flooded limestone forest, dry limestone forest and secondary forest (dipterocarp forest that had been selectively logged many years ago) (Table 1; Fig. 1). The *Macaranga* species examined were *M. pruinosa* (Miq.) Mull.Arg., *M. hypoleuca* (Reichb.f. & Zoll.) Műll.Arg. and *M. triloba* (Thunb.) Müll.Arg. (family Euphorbiaceae). A further study site in North Selangor Peninsular Malaysia (Table 1) was chosen to collect samples of *M. pruinosa* for the laboratory leaf leaching and seedling absorption experiments (Studies 2 and 3).

**Table 1:** Description and location of study sites

Location	Coordinates	Description
Mulu National Park, Sarawak, East Malaysia		
Peat swamp forest	N04°02′50.2″ E114°48′56.1″	Pristine TPSF. Peat substrate (1.5 m deep) overlying a substrate of river pebbles (formed on a cut-off river meander), blackwater, high water table (30 cm above forest floor). No streams flow in or out, but water table rises and falls seasonally
Freshwater swamp forest	N04°02′11.62″ E114°48′06.95″	Similar vegetation to TPSF. Clay and sand substrate. Water table clear, 30 cm above forest floor. Connected to main river by a small stream
Dry limestone forest	N4°01′27.18″ E114°49′11.21″′	Dipterocarp forest (*Hopea andersonii* and *Shorea multiflora* common) on limestone hill next to a river. Substrate of sand, clay and limestone
Flooded limestone forest	N4°3′26.48″ E114°49′38.08″	Dipterocarp forest in a depression next to a river. Substrate of sand, clay and limestone. Water table 25 cm above surface
Secondary forest	N04°02′59.59″ E114°48′54.98	Secondary forest on mineral soil. Dipterocarp forest which had been logged prior to gazettement of Mulu National Park
Peat Swamp Forest, Peninsular Malaysia		
North Selangor	N3°39′30.8″ E101°19′18.4″	Mixed TPSF. Selectively logged ~ 25 years previously in 1980s. Canopy height up to 30 m. Maximum peat depth 15 m

#### Study 1: Effect of waterlogging and acidity on phenolic compounds in species of *Macaranga* in different forest types

##### Field sampling

At each study site in Gunung Mulu National Park, Sarawak (Fig. 1), dissolved oxygen, temperature and pH of the water were measured using a Eutech Cyberscan PD650 m. Water depth of the flooded sites (TPSF, Flooded Limestone Forest and Freshwater Swamp Forest) was measured using a weight on a rope which was lowered until it reached the bottom and the length of the wet rope was measured. To determine the pH of the substrate, 10 g of the substrate was mixed with 50 mL of 5% CaCl_2_ solution and shaken for 30 min. The pH was then measured using a pH probe. The study was conducted over 1 year and samples were collected every 90 days. From each site, five trees of *M. pruinosa, M. hypoleuca* and *M. triloba* were selected and tagged. Three mature leaf samples were collected from each of the five trees of each species at each site (N = 15 samples/species/site). Extraction and analyses of phenolic compounds from the leaf samples are described below.

#### Study 2: Leaching of phenolic compounds from senescent leaves

A leaching experiment was conducted to determine the concentration of phenolic compounds leaching from senescent *Macaranga pruinosa* leaves (collected from North Selangor Peat Swamp Forest: Table 1) into distilled water. Five replicates of 10 g of freshly senescent leaves were cleaned and then soaked in 500 mL of distilled water for a period of 13 days. The leachate was removed and replaced with another 500 mL of distilled water at 0.5, 1, 2, 3, 4, 5, 6, 7, 9, 10, 11 and 13 days. Triplicates of ~ 5 mL of leachate were removed at each time and filtered through a 0.45 µm RC membrane filter. The filtrate was then analysed for TPC and TTC (see below).

#### Study 3: Absorption of phenolic compounds by tree seedlings

Seedlings of TPSF *M. pruinosa* were propagated by the grafting method. An injury was made to the joint of a young branch or stem (collected from North Selangor TPSF), topsoil (black soil) was then wrapped around the injured part using cheesecloth. They were then allowed to root for 2 weeks before being transplanted to a bag containing 500 g of peat (collected from North Selangor Peat Swamp Forest). Each bag was sealed and the water table was maintained at 1.5 cm above the soil surface. The seedlings were watered with 50 mL of rain water every 2 days, and were exposed to sunlight from 7 a.m. to 6 p.m. daily depending on weather conditions. When the seedlings had grown to approximately 50 cm, they were moved to the Monash University Green House in Kuala Lumpur. A total of 45 plants were used for each experiment. Eight treatments were used for the experiment (Table 2). Each bag was treated with phenolics at 10 mg/100 g of fresh peat. For each treatment, the flavonoid combinations were dissolved in 10 mL of water before being added directly into the soil.

**Table 2:** Summary of treatments used to study phenolic uptake by seedlings

Treatment	Quantity
Control	Nothing added
Ferulic acid	10 mg/100 g
*p*-Coumaric acid	10 mg/100 g
Kaempferol	10 mg/100 g
Taxifolin	10 mg/100 g
Quercetin	10 mg/100 g
Phenolic acids	Mixture of ferulic acid and *p*-coumaric acid (5 mg/100 g of each)
Flavonoids	Mixture of kaempferol, taxfolin and quercetin (2.5 mg/100 g of each)

Two leaf samples were collected from each plant at days 0, 1, 2, 3, 4, 5, 10, 15, 20, 25 and 30. Leaf samples were extracted with 100% methanol as described for Study 1. Analyses of TPC of the methanolic leaf extracts were performed and quantification of ferulic acid, *p*-coumaric acids, kaempferol and quercetin are described below. This experiment was performed twice to verify the data.

RP-HPLC (reversed phase high performance liquid chromatography) was used to quantify the concentrations of the compounds in the leaf extracts over a period of 30 days in order to determine if the plants are (i) capable of directly absorbing them via the root system or (ii) produce these compounds in the leaves or (iii) use a combination of both. RP-HPLC analysis was conducted on the same day on which the leaves were collected.

### Laboratory analyses

#### Sample preparation

Leaf extracts were prepared and analyzed as in Lim et al. [9, 10] and Yule et al. [6] as follows: 1 g of each of the fresh leaf samples (in triplicate) was crushed mechanically into powder in liquid nitrogen. The crushed samples were extracted with 50 mL of 100% methanol by shaking the suspension on an orbital shaker for 1 h and then the extracts were filtered under suction. The extracts were stored at − 20 °C until further use.

#### Total phenolic content (TPC)

To determine the total phenolic content (TPC), the Folin Ciocalteu assay [16] was used. Samples (300 μL, triplicate) were placed in test tubes followed by 1.5 mL of Folin Ciocalteu’s reagent (10× dilution) and 1.2 mL of 7.5% sodium carbonate. Tubes were left to stand in the dark for 30 min prior to measurement of absorbance at 765 nm. Total phenolic content was expressed as gallic acid equivalent (GAE) in mg GAE/100 g material. The calibration equation was y = 0.0111x − 0.0148 (r^2^ = 0.9998) where y = absorbance and x = concentration of gallic acid in mg/L.

#### Total flavonoid content (TFC)

Total flavonoid content was determined using the aluminium chloride colorimetric method described by Chang et al. [17]. 0.5 mL of sample was diluted with 1.5 mL of methanol. 100 μL of 10% AlCl_3_, 0.1 mL of 1.0 M potassium acetate as well as 2.8 mL of distilled water was added into the extract solution. The final volume was 5.0 mL. This mixture was incubated for 30 min at room temperature. The absorbance of the extracts was measured at 435 nm against distilled water. For the blank, the 0.1 mL of 10% AlCl_3_ was substituted with distilled water. TFC values were expressed as quercetin equivalent in mg per 100 g of material. The calibration equation used was y = 0.0686x + 0.001 (R^2^ = 0.9984) whereby x represents the concentration in mg/L and y represents the absorbance at 435 nm.

#### Total tannin content (TTC)

Determination of total tannin content was conducted using a similar method for the determination of TPC described by Makkar et al. [18]. Briefly, 0.3 mL of extract was mixed with 1.5 mL (1:10) Folin Ciocalteu’s (F&C) reagent and 1.2 mL of 20% (w/v) sodium carbonate. The solution was allowed to stand for 30 min in the dark. The absorbance measured at 765 nm allowed the determination of TPC in terms of tannic acid equivalent (TAE). In a separate tube, 100 mg of PVPP (polyvinylpolypyrrolidone) was weighed and added to a mixture of distilled water and extract (1.0 mL each). The mixture was then vortexed at 4 °C for 15 min and centrifuged for 10 min at 3000*g*. The supernatant which contained only simple phenolics as tannins were bound to PVPP was collected. The phenolic content of the supernatant was then determined using the F&C reagent. From the results, tannin content of the sample was calculated as: Total phenolics − non-tannin phenolics = tannins (mg TAE/100 g). TTC was expressed as TAE in mg per 100 g of materials. The calibration equation was y = 0.097x − 0.0012 (R^2^ = 0.998) whereby x represents concentration in mg/L and y represents absorbance at 765 nm.

#### High performance liquid chromatography (HPLC)

Crude extracts were dissolved as much as possible in 1 mL of 30% methanol (sonicated). Then, 1 mL of hexane was added to each fraction, vortexed and allowed to stand for 1 min. Fractions were centrifuged for 30 s. The hexane layer (top layer) which contained fatty compounds was removed and the entire process was repeated twice. The aqueous layer was filtered through a membrane filter (pore size 0.45 µm) prior to injection into the HPLC for chromatographic analysis. The HPLC consisted of quaternary vacuum degasser pump and diode array detector (Agilent 1200 Series). Samples were injected through a Rheodyne manual injector valve fitted with 20 μL sample loop. The column consisted of a phenyl-bound silica column (100 × 4.6 mm; 5 µm particle size) from Thermo Scientific.

Gradient mode was used in this analysis, involving two solvents: (1) 100% methanol and (2) water, acidified to approximately pH 2.5 with 0.1% trifluoroacetic acid. The elution profile was 40% mobile phase 1–60% mobile phase 2 in a linear gradient from 0 to 20 min. The flow rate was set at 1 mL/min. The detection wavelengths used were 210, 245, 280 and 365 nm with reference wavelength set at 700 nm.

#### Identification and quantification of phenolic compounds

Specific phenolic compounds in the leaves of *M. pruinosa* were identified based on (i) retention time, (ii) spiking with known standard and (iii) comparing the absorbance spectra of both spiked and existing compounds. The concentration of each compound was determined based on the construction of a standard curve for each of the phenolic compounds identified. A total of four phenolic compounds were identified from the leaves and four different standard curves were constructed. The standard phenolic compounds were purchased from Sigma-Aldrich. The content of each compound was expressed as mg/100 g of fresh leaves.

#### Statistical analyses

For comparison between the mean concentrations of phenolic compounds in the mature leaves of *M. pruinosa*, *M. hypoleuca* and *M. triloba* with respect to forest type, one way analysis of variance (ANOVA) were used and significant differences were identified using the Tukey’s HSD (honestly significant difference) test. Statistical analyses were conducted using SPSS version 16.0 and differences were considered to be significant at P < 0.05.

## Results

### Study 1: Effect of waterlogging and acidity on phenolic compounds in species of *Macaranga* in five different forest types

Five forest types, three flooded and two dry land, were compared in Mulu National Park, Sarawak. The TPSF was flooded with acidic water (pH 3.12) with very high levels of phenolic compounds (354 ± 24 GAE/100 g) in comparison with the other forest types which all had neutral to alkaline soils and water, and soil TPC several times lower (31.2–132 GAE/100 g) (Table 3).

**Table 3:** Summary of chemical and physical properties of each type of forest substrate

Parameters	Peat swamp forest	Freshwater swamp forest	Flooded limestone forest	Dry limestone forest	Secondary forest
TPC (mg GAE/100 g) of soil (Mean ± 1 S.E.)	354 ± 24	132 ± 11	57 ± 0.2	31.2 ± 0.1	66 ± 0.7
Soil pH	3.17	6.87	8.73	8.37	6.34
Water table (cm above surface)	28–40	22–30	20–25	0	0
Water pH	3.12	6.32	8.83	–	–

**Fig. 2:**
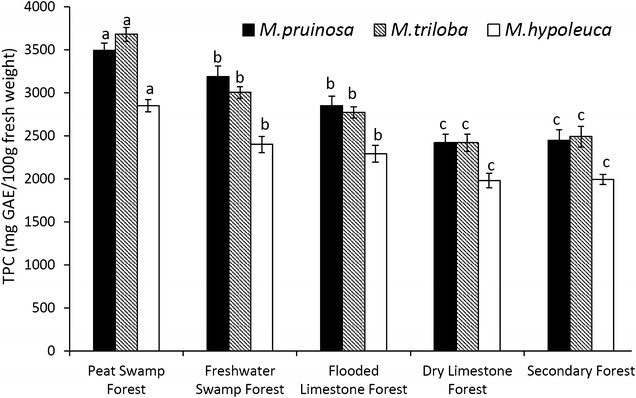
Mean TPC values of mature *Macaranga* leaves in Mulu National Park (± 1 S.D., N = 20). For each species, values followed by the same letter (a, b, c) are not significantly different between forest types at P < 0.05 (Tukey HSD test)

For each of the three *Macaranga* species, TPC values were significantly highest in the TPSF (e.g. 3680 ± 82.5 GAE/100 g for *M. triloba*) followed by the freshwater swamp forest (3190 ± 66 GAE/100 g for *M. triloba*) and the flooded limestone forest (2772 ± 68 GAE/100 g for *M. triloba*) (Fig. 2). TPC values of leaves from all the flooded sites were significantly higher than the two dry land sites, which did not show any significant difference (2418 ± 100 for dry limestone forest and 2410 ± 118 GAE/100 g for secondary forest for *M. triloba*). Thus the highest TPC values were promoted by a combination of acidity (in the TPSF) and waterlogging (in all the flooded forests), but waterlogging alone also promoted an increase in foliar TPC (Table 3, Fig. 2).

**Fig. 3:**
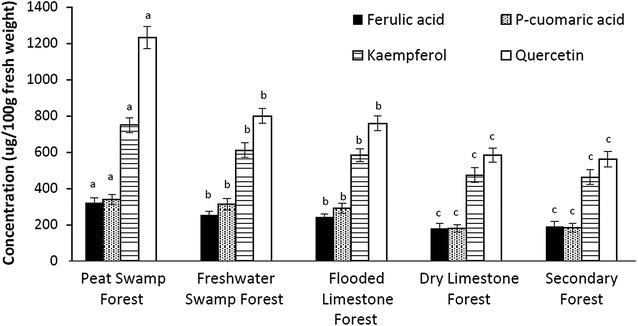
Mean concentrations (± 1 S.D., N = 20) of phenolic compounds in the mature leaves of *M. pruinosa* with respect to forest type. For each phenolic compound values followed by the same letter (a, b, c) are not significantly different at P < 0.05 (Tukey HSD test)

The concentrations of ferulic acid, *p*-coumaric acid, kaempferol and quercetin in the mature leaf extracts from *M. pruinosa* trees from the five forest types (Table 3) were also determined to investigate the effect of flooding and acidity (Fig. 3). Trends in concentrations of all four compounds reflected those of leaf TPC values (Fig. 2). Concentrations of the four known compounds were significantly higher in the plants collected from the TPSF, followed by those collected from the freshwater swamp forest and flooded limestone forest which in turn were significantly higher than the dry land forests. In each forest site, quercetin had the highest concentrations, supporting the suggestion that *M. pruinosa* has the capacity to absorb it via the roots and synthesize it from low molecular weight phenolic acids.

### Study 2: Leaching of phenolic compounds from senescent leaves

**Fig. 4:**
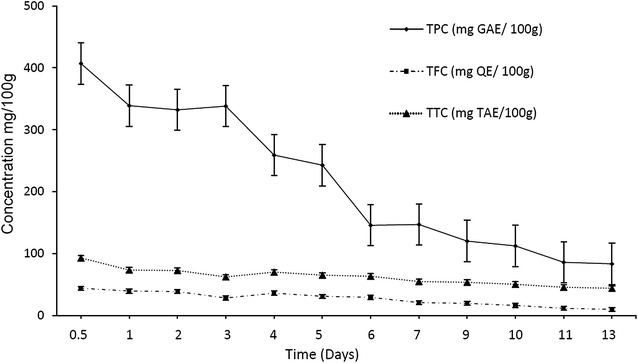
Mean TPC, TFC and TTC (± 1 S.D., N = 5) leached from senescent *M. pruinosa* leaves over time

Phenolic compounds leached rapidly from *M. pruinosa* leaves upon immersion in distilled water (Fig. 4). The leaching of a large proportion of phenolic compounds, occurred within 48 h with a further rapid decrease until Day 6 (120 h) after which, leaching continued slowly until Day 13 (312 h) (Fig. 4). This is typical of leaching of senescent leaves which generally peak at 24–48 h after immersion, with further leaching continuing for weeks [19].

Although using peat water instead of distilled water might be expected to influence leaching, a similar result was reported by Yule and Gomez [11] who measured levels of dissolved organic carbon leached from *M. pruinosa* and two other TPSF species in both Milli-Q and peat water. They found no significant difference between leaching of TPSF plants in Milli-Q water and peat water.

### Study 3: Absorption of phenolic compounds by tree seedlings

**Fig. 5:**
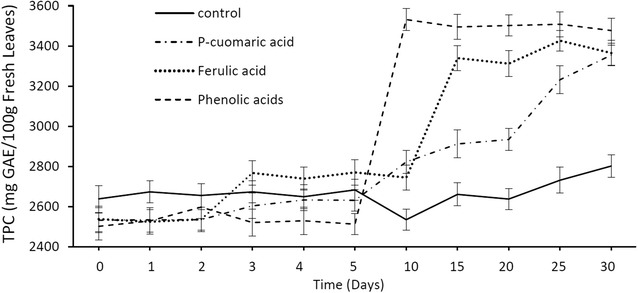
Mean TPC values (± 1 S.D., N = 45) of *M. pruinosa* leaf extracts from seedlings treated with phenolic acids

Mean TPC values of methanolic leaf extracts of *M. pruinosa* seedlings treated with phenolic acids showed a strong increase from day 5 (phenolic acids and *p*-coumaric acid) and day 10 (ferulic acid) onwards (Fig. 5). Seedlings subjected to the control treatment (no phenolics added) had stable TPC values up to day 20 after which the value increased slowly to day 30, indicating some synthesis of phenolic compounds within the plants. Comparison of the controls with the treated seedlings demonstrates that uptake of phenolic compounds by the seedlings is a greater source of the compounds than synthesis within the plants.

**Fig. 6:**
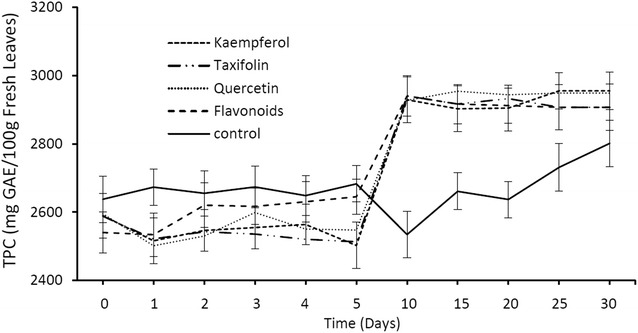
Mean TPC values (± 1 S.D. N = 45) of *M. pruinosa* leaf extracts from seedlings treated with flavonoids

Seedlings treated with flavonoids (Fig. 6) showed a similar response to those treated with phenolic acids (Fig. 5). An increase in TPC values commenced on day 5 which then stabilized on day 10. Generally, seedlings treated with phenolic acids (ferulic acid, *p*-coumaric acid and a combination of both) showed higher TPC values compared to plants treated with flavonoids (kaempferol, taxifolin, quercetin and combination of all flavonoids). It is likely that these plants have a higher affinity to absorb phenolic acids than flavonoids available in the soil/water due to the simpler molecular structure and lower molecular weight of phenolic acids. The absorbed phenolic acids and flavonoids would then be transported to the leaves to supplement the production of phenolic compounds via the flavonoid pathway in which phenolic acids are converted to flavonoids and these are in turn converted to tannins (proanthocyanidins) [20] (Fig. 8). An important intermediate for the synthesis of flavonoids from phenolic acids is naringenin. Phenolic acids (ferulic acid and *p*-coumaric acid) are initially converted into naringenin (Fig. 8) which also acts as the precursor for the formation of kaempferol which is converted directly to proanthocyanidins (tannins) or taxifolin.

### Variation of phenolic acids and flavonoids with time

**Fig. 7:**
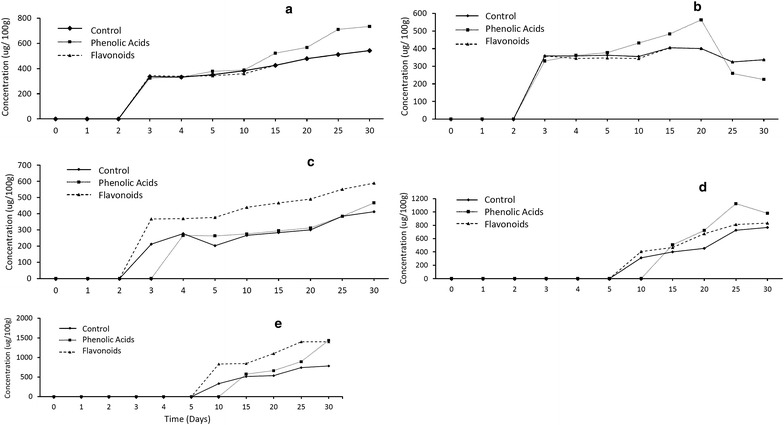
Variation of phenolic acids and flavonoids in leaves of *M. pruinosa* seedlings with time following additions of **a** *p*-coumaric acid, **b** ferulic acid, **c** naringenin, **d** kaempferol and **e** quercetin (N = 30)

**Fig. 8:**
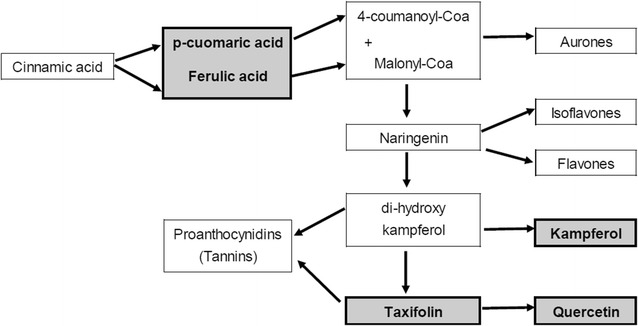
A partial section of the flavonoid pathway showing the formation of compounds of interest (modified from Grotewold and Rausher [20]). Compounds highlighted were identified from TPSF *M. pruinosa* leaves

Concentrations of phenolic compounds in the leaf extracts were quantified over a period of 30 days to determine if the plants are (1) capable of directly absorbing phenolic compounds via the root system or (2) produce phenolic compounds in the leaves or (3) use a combination of both (Fig. 7a–c). The addition of phenolic acids caused an increase in flavonoids: *p*-coumaric acid concentrations from day 2 onwards (Fig. 7a), and ferulic acid increased from day 5 to day 15 after which the concentrations steadily decreased (Fig. 7b). However, the steady decrease of ferulic acid paralleled an increase in the production of both kaempferol and quercetin. This suggests the addition of phenolic acids promoted the production of flavonoids in the treated seedlings that is consistent with the conversion mechanism depicted in the flavonoid pathway (Fig. 8). However, in the flavonoid treatment, the production of flavonoids began on day 10. It was observed that the addition of flavonoids into the soil also increased the production of flavonoids in the treated plants but the increase only began after day 20 (Fig. 7c). Quercetin and kaempferol showed a marked increase on day 25 (Fig. 7d, e). A possible explanation could be that the increase of these two compounds is a natural response of the TPSF plants to combat herbivory via both synthesis from low molecular weight phenolics, particularly ferulic acid, and also by direct absorption from the soil. This hypothesis is supported by the finding that concentrations of phenolic acids increased up to day 4, remained stable until day 20 and then decreased as flavonoid levels begin to increase. In the treatment whereby both flavonoids and phenolic acids were added, production of quercetin was promoted. These findings can explain the high concentrations of quercetin in mature TPSF *M. pruinosa* leaves [10].

**Fig. 9:**
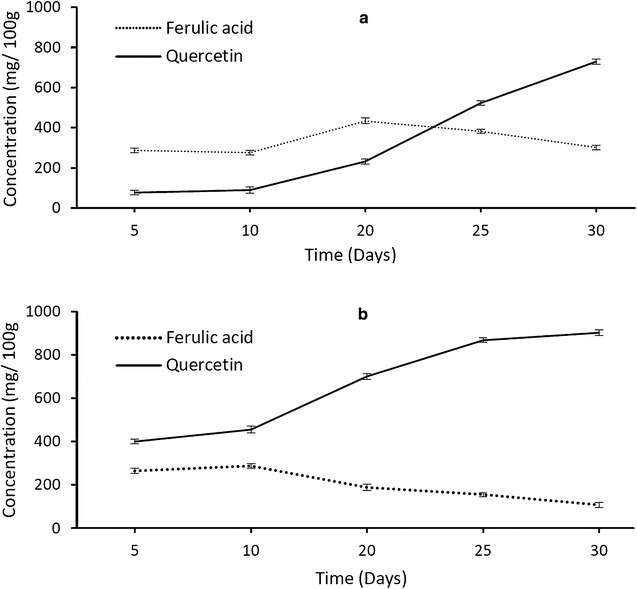
Mean concentrations (± 1 S.D. N = 30) of ferulic acid and quercetin in **a** seedlings treated with ferulic acid only and **b** treated with quercetin only

Seedlings treated with ferulic acid showed an increase in ferulic acid concentration which peaked at the 20th day and subsequently decreased (Fig. 9a). While the concentration of ferulic acid was decreasing, the concentration of quercetin increased. This strongly suggests that ferulic acid is being converted to quercetin which correlates to the phenylpropanoid metabolic pathway as shown in Fig. 8. In Fig. 9b, the seedlings were only treated with quercetin. By comparing the quercetin values in Fig. 9a, b, the initial concentration in seedlings provided with solubilized quercetin was higher. Also noticeable was that the concentration of quercetin increased faster than when only ferulic acid was provided. This indicates that TPSF *M. pruinosa* seedlings are capable of absorbing the solubilized phenolic compounds into their system and using the compounds to supplement the precursors needed for the production of flavonoids.

## Discussion

### Study 1: Effect of waterlogging and acidity on phenolic compounds in species of *Macaranga* in five different forest types

The five forest types in Mulu National Park, Sarawak differed significantly with respect to flooding, acidity and levels of phenolic compounds (Table 3). Acidity and levels of phenolic compounds were highest in the TPSF whereas the other forest types had neutral to alkaline soils and water, and soil TPC several times lower. Similarly, TPC values for each of the three *Macaranga* species were significantly higher in the flooded sites (being highest in the TPSF) than the two dry land sites. This shows that a combination of acidity (in the TPSF) and waterlogging (in all the flooded forests) promoted TPC, but waterlogging alone also promoted an increase in foliar TPC (Table 3, Fig. 2). Whether acidity also promotes absorption of phenolics is not proven as it is likely that the higher concentrations of phenolic compounds in peat water (which result in acidity) enable a higher rate of absorption of these compounds. Concentrations of ferulic acid, *p*-coumaric acid, kaempferol and quercetin in the mature leaf extracts from *M. pruinosa* reflected those for TPC, being significantly higher in the plants collected from the TPSF, followed by those collected from the freshwater swamp forest and flooded limestone forest, then the dry land forests. Quercetin had the highest concentrations in all sites, supporting the suggestion that *M. pruinosa* has the capacity to absorb it via the roots and synthesize it from low molecular weight phenolic acids. Ferulic acid, *p*-coumaric acid, kaempferol, taxifolin and quercetin have anti-herbivory and anti-lipid peroxidation properties [5] which would be of particular benefit to the plants given the extreme, low nutrient conditions of the TPSF where protection of leaves would be favoured over new growth [11].

The significantly high levels of phenolics in the substrate and water are the key difference between peat swamp forest and freshwater swamp forest. In TPSF, water only flows out of the swamps (depleting nutrients), whereas in freshwater swamps, water flows in bringing nutrients. This factor influences the availability of phenolic compounds through its effects on plant adaptations to low nutrients (tough, phenol rich, toxic leaves) which in turn affect litter decomposition and leaching [11, 12]. This leads to the questions—are phenolic compounds leached from leaves a potential source of phenolics for trees (Study 2) and can plants absorb phenolic compounds via their roots (Study 3)?

### Study 2: Leaching of phenolic compounds from senescent leaves

Immersion of *M. pruinosa* leaves in distilled water resulted in rapid leaching of phenolic compounds (Fig. 4). The results indicate that, most phenolic compounds leached out from fallen leaves within 2 days in the PSF making it available for uptake by the plant roots and the initial levels of tannins leaching from leaves were similar to those determined for surface peat [10] indicating that leaching of leaf litter is probably the major source of tannins on the forest floor. Leaves may contain up to 40% tannin by dry weight and although bark and wood can also contain high levels of tannins, these decompose more slowly than leaves and would not be as susceptible to leaching [13]. Furthermore, although litter fall in TPSF may have peaks related to climate it typically occurs throughout the year [21], providing a constant supply of phenolic compounds.

Apart from phenolic compounds, leachates include amino acids, sugars and other soluble compounds, and as the leaves are decomposed by fungi and bacteria, leaching is enhanced [19], yet despite 13 days of immersion, there were no physical changes (e.g. skeletonized leaves) observed in the leaves, indicating that little microbial decomposition occurred. Ong et al. [12] measured nutrient release during leaching of TPSF leaves from the same site as the present study and found that although there was a significant loss of phosphorus, there was less nitrogen lost, and very small loss of carbon, since the leaves barely decomposed. Yule and Gomez [11] used *M. pruinosa* and two other TPSF leaf species from the same site, and showed that leaching of all species resulted in initial rapid weight loss but this loss was slower than that reported for temperate leaf species by Petersen and Cummins [22] and for most native leaves from tropical forests on mineral soil reported by Boyero et al. [23]. This was due to the slower microbial breakdown of TPSF leaves resulting from their increased toughness and high levels of phenolic compounds. This difference in the leaching and decomposition of leaves from TPSF and leaves from other ecosystems is crucial in the accretion of peat and sequestration of carbon in TPSF.

### Study 3: Absorption of phenolic compounds by tree seedlings

As shown in study 1 and Yule et al. [6], TPSF are distinguished by the high levels of phenolic compounds which darken their waterlogged substrate of peat. Our results demonstrate that *M. pruinosa*, a common TPSF tree, is capable of a direct uptake of phenolic compounds (both phenolic acids and flavonoids) via the root system, which supports the previous findings of Lim et al. [10], in which there were high concentrations of lower molecular weight phenolics detected in the fine and thick roots of TPSF *M. pruinosa.* When *M. pruinosa* seedlings were provided with phenolic acids and flavonoids, levels of TPC in the leaves increased much more rapidly and strongly than in the controls (no added compounds) demonstrating that uptake of phenolic acids and flavonoids by the seedlings is a greater source of the compounds than synthesis within the plants, since the controls indicated only modest and more delayed synthesis of phenolic compounds (Figs. 5, 6). Seedlings treated with phenolic acids (ferulic acid, *p*-coumaric acid and a combination of both) showed higher TPC values compared to plants treated with flavonoids (kaempferol, taxifolin, quercetin and a combination of all flavonoids) (Fig. 6), presumably reflecting the simpler molecular structure and lower molecular weight of phenolic acids compared with flavonoids which facilitated their uptake via the roots.

The addition of phenolic acids caused an increase in *p*-coumaric acid from day 2 onwards (Fig. 7a). Although ferulic acid concentrations initially increased they steadily decreased after day 20 (Figs. 7b, 9a) paralleling an increase in the concentrations of both kaempferol and quercetin (Figs. 7d, e, 9a). This suggests that the phenolic acids were converted to kaempferol and quercetin as depicted in the flavonoid pathway (Fig. 8). When seedlings were provided with quercetin (Fig. 9b) the concentration of quercetin in the leaves increased more rapidly and to a higher level than when ferulic acid was added (Fig. 9a) indicating that TPSF *M. pruinosa* seedlings are capable of absorbing the solubilized phenolic compounds via their roots and using the compounds to supplement the precursors needed for the production of flavonoids.

Following absorption, the phenolic acids would be transported to the leaves where they would be initially converted into naringenin (Fig. 7c) which also acts as the precursor for the formation of kaempferol (Fig. 7d) which is converted directly to proanthocyanidins (tannins) or taxifolin via the flavonoid pathway (Fig. 8) [20]. Synthesis of phenolic compounds in the leaves is supported by the observations of Lim et al. [10] who found that relative concentrations of low (phenolic acids, flavonoids) to high (tannins and derivatives) molecular weight phenolics decreased as leaves matured indicating conversion within leaves. There have been no previous studies conducted to determine the uptake of phenolic compounds via direct absorption by roots although there have been studies suggesting the presence of simple phenolics (e.g. caffeic acid, *p* coumaric acid and ferulic acid) and other precursors that could be used for the formation of these compounds [20].

Results from this study suggest that rather than being stressful for plants, the phenolic-rich acidic water of TPSF provides the opportunity for plants to recycle both low (phenolic acids) and relatively high (flavonoids) molecular weight phenolic compounds and to use phenolic acids to synthesize flavonoids which can then be used in defense or other metabolic activities. In study 3, TPSF seedlings were shown to be capable of converting phenolic acids (i.e. ferulic acid and *p*-coumaric acid) into more complex phenolic compounds (i.e. kaempferol and quercetin). This supports the results of our earlier study [10] which showed that the relative concentrations of low (phenolic acids, flavonoids) to high (tannins and derivatives) molecular weight phenolics in *M. pruinosa* decreased as leaves matured indicating conversion within leaves. This suggests that young leaves either, are capable of producing low molecular weight simple phenolic compounds (phenolic acids and flavonoids), and/or the plants absorb these from the peat substrate. Phenolic acids confer initial chemical protection for the young leaves which are softer and so have less physical protection against herbivory, UV and other factors. It seems probable that, as the leaves mature, these simple low molecular weight phenolics are converted to more complex phenolics following the biosynthetic pathway outlined by Niesh [24] and Schijlen et al. [25]: phenolic acids → flavonoids (or isomers) → tannins (both condensed and hydrolysable).

Production of phenolic compounds in plants in response to stress such as herbivory or pathogen attack is typically considered to be a trade-off at the expense of plant growth and reproduction [5]. This is predicted by resource-based allocation theory whereby allocation of carbon for secondary metabolism (defense) is at the expense of primary metabolism (growth and reproduction) [26, 27]. In the nutrient limited TPSF, the ability of plants to reabsorb and thus to recycle phenolic compounds and to synthesize high molecular weight compounds within the leaves for defense against herbivory and pathogen attack would thus be an adaptation to survive which would be enhanced by the acid, waterlogged conditions. This explains our earlier results [6, 9] where we showed that the concentration of phenolic compounds was higher in various ferns, gingers and trees found in TPSF compared with the same species growing on dry land mineral soils. For those plants that possess the required adaptations to survive waterlogging, the conditions of TPSF may not be stressful after all, but may facilitate the uptake and synthesis of protective phenolic compounds which are key to peat swamp development and maintenance.

The high levels of phenolic compounds that are typical of leaves of TPSF plants inhibit decomposition, and decrease the water and soil pH and make them toxic, creating the conditions necessary for peat accretion. Since carbon in TPSF is sequestered largely as phenolic compounds (lignin, tannins, humic acids and other plant secondary compounds) which form the peat substrate, colour and acidify the water, and give structure and protection to the plants, the fact that phenolic compounds leached from leaves can be recycled by plants through reabsorption by roots and then used in further synthesis of phenolic compounds, is clearly important in the formation and maintenance of peat swamp ecosystems.

Our results help to explain how the regional peat swamp forests of the Indo-Malaysian regions can support such diverse, distinctive and productive forests despite the extreme conditions and exceptionally slow rates of litter and nutrient recycling. Furthermore it explains how draining the peat, increasing the pH and clearing the vegetation will adversely impact peat accretion and carbon sequestration through impeding recycling of phenolic compounds.

## Conclusions

The high levels of phenolic compounds in the lignin dominated peat substrate and in the leaves of TPSF plants distinguish TPSF from dryland forests and degraded peat lands, and as shown in this study, they also distinguish TPSF from other flooded forests—freshwater swamp forests and flooded limestone forests. Phenolic compounds leach rapidly from senescent *M. pruinosa* leaves and initial levels of tannins leaching from leaves were similar to those determined for surface peat and thus fallen leaves are probably the major source of phenolic compounds in the water of TPSF. These phenolic compounds acidify the water and impede decomposition of organic matter leading to peat accretion. They are then available to be recycled back into the plants and used in the accumulation and synthesis of high molecular weight phenolics which would be valuable for defense against herbivory and microbial infections in the nutrient poor environment, and will further impede microbial decomposition of senescent leaves. Consequently phenolic compounds are fundamental to the extreme conditions of TPSF with respect to acidity and impeded decomposition and hence they are key to TPSF development and maintenance and thus the sequestration of carbon. Conversion to oil palm and other activities resulting in the drainage and degradation of TPSF would thus reverse the conditions necessary for retardation of decomposition, accumulation of peat and sequestration of carbon.

## Abbreviations

GAE: gallic acid equivalent
PVPP: polyvinylpolypyrrolidone
RP-HPLC: reversed phase high performance liquid chromatography
TAE: tannic acid equivalent
TFC: total flavonoid content
TPC: total phenolic content
TPSF: tropical peat swamp forests
TTC: total tannin content
